# Nonlinear phenomena in marmot alarm calls: a mechanism encoding fear?

**DOI:** 10.1098/rstb.2024.0008

**Published:** 2025-04-03

**Authors:** Daniel T. Blumstein

**Affiliations:** ^1^ Department of Ecology and Evolutionary Biology, University of California, Los Angeles, CA 90095-1606, USA; ^2^ The Rocky Mountain Biological Laboratory, Crested Butte, CO 81224, USA

**Keywords:** alarm communication, fear, nonlinear vocal phenomena, yellow-bellied marmots

## Abstract

I review a case study of marmots that contributed to the empirical basis of the nonlinearity and fear hypothesis, which explains why certain nonlinear acoustic phenomena (NLP) are produced in extremely high-risk situations and communicate high urgency. In response to detecting predatory threats, yellow-bellied marmots (*Marmota flaviventer*) emit alarm calls and, in some situations, emit fear screams. Prior work on marmots has shown that call production is associated with the degree of risk the caller experiences and that they are individually distinctive. Receivers respond to calls and are sensitive to variation in caller reliability. Calls also contain nonlinear acoustic phenomena. Work has shown that socially isolated animals and those infected with *Eimeria*, an intestinal parasite, produced ‘noisier’ calls. However, animals that were likely under greater stress (as measured with faecal glucocorticoid metabolites) produced more structured and less noisy calls. The addition of NLP increases responsiveness in receivers. NLP in alarm calls have modest heritability. Taken together, the study of NLP in marmots has enhanced our understanding of the potential information encoded in alarm calls and is consistent with the hypothesis that variation in NLP production communicates fear, which stimulates work with other species, including humans.

This article is part of the theme issue ‘Nonlinear phenomena in vertebrate vocalizations: mechanisms and communicative functions’.

## 1. Introduction

Upon detecting predators, many species produce specific vocalizations, referred to as alarm calls [1]. These calls may be targeted to conspecifics to create pandemonium, which may make it more difficult for a predator to target a prey [2,3] or to warn them of the presence of the predator [4]. They also may be directed to heterospecific prey to create pandemonium or to warn them of the presence of the predator or to the predators themselves to signal their detection and to potentially discourage pursuit [5]. The structure of alarm calls varies by species, and there is some evidence that they have evolved to be either particularly detectable or less detectable to predators. For instance, chickens (*Gallus gallus*) produce lower-frequency, rapidly paced calls in response to detecting lower-risk mammalian predators. The production of these terrestrial alarm calls is not influenced by having other conspecifics nearby. In contrast, chickens are more likely to emit alarm calls in response to a relatively high-risk raptor when others are around. Importantly, these calls are of relatively high frequency and fade in and out, making them more cryptic. These calls are likely designed to alert conspecifics, which freeze in response to hearing them [6,7].

A specific type of predator-elicited vocalization that some species produce is referred to as fear scream [8,9]. Fear screams, as Darwin [10] noted, are cries of assistance, given by young to solicit aid from their parents, as seen in piglet (*Sus scrofa*) screams [11]. In the context of predator-elicited calls, such screams are relatively rarely given, compared with more traditional alarm calls given in response to detecting a predator, and the structure of these screams is remarkably similar across species. Hypothesized functions of fear screams include soliciting aid from conspecifics [12], warning kin [13], startling the predator, permitting the screamer to escape [14] and/or functioning to attract other predators to encourage competition among predators, which may permit the prey to escape [8]. Importantly, these need not be mutually exclusive functions.

The nonlinearity and fear hypothesis [15,16] posits that highly aroused animals produce nonlinear vocalizations because they lose control of their larynx [17] and ‘over-blow’ their vocal production apparatus. However, nonlinear acoustic phenomena (NLP) may also be produced when animals lose vocal control because they become fatigued by producing long vocalizations, as seen in indris (*Indri indri*) [18,19]. And, as Rendall [20] argues in this issue, the production of NLP may be intentional to evoke specific responses in perceivers.

We now have considerable evidence from a variety of species that suggests that NLP communicate urgency and capture the attention of perceivers. For instance, meerkats (*Suricata suricatta*) vary the structure of their functionally referential alarm calls as a function of urgency: calls elicited in more urgent situations contain more of what appears to be deterministic chaos (a type of NLP) and are ‘noisier’ [21]. NLP are produced in stressful situations in horses [22] and they are important cues in communicating arousal and valence to other horses [23]. The occurrence of deterministic chaos in dog (*Canis familiaris*) whines is associated with stressful situations, including separation from their human caretakers [17]. Massenet *et al*., in this issue [24], note that the production of puppy NLP increases with the time since separation from their mother and provides vocal cues to high arousal. Humans are able to detect the emotional context of a variety of animal vocalizations [25]. Recent work has shown that the addition of NLP in puppy whines increases human perceptions of distress [26]. Humans are also able to perceive distress in bonobo (*Pan paniscus*) and chimpanzee (*Pan troglodytes*) distress vocalizations, which are characterized by some NLP [27]. The addition of NLP to human non-verbal sounds (yells and moans) increased perceived aversiveness and emotional intensity [28]. NLP capture attention, as seen in koala (*Phascolarctos cinereus*) rejection calls [29] and in red deer (*Cervus elaphus*) sexual calls [30].

Here, I review what is known about alarm calling in yellow-bellied marmots (*Marmota flaviventer*) and the research in birds, lizards and humans. My marmot studies were stimulated by this work and together helped develop the nonlinearity and fear hypothesis. I will start by describing the marmot system and then talk about how fear screams and alarm calls contain nonlinearities and the conclusions we have drawn about putative causality.

## 2. The marmots of the Rocky Mountain Biological Laboratory

**Figure 1.**
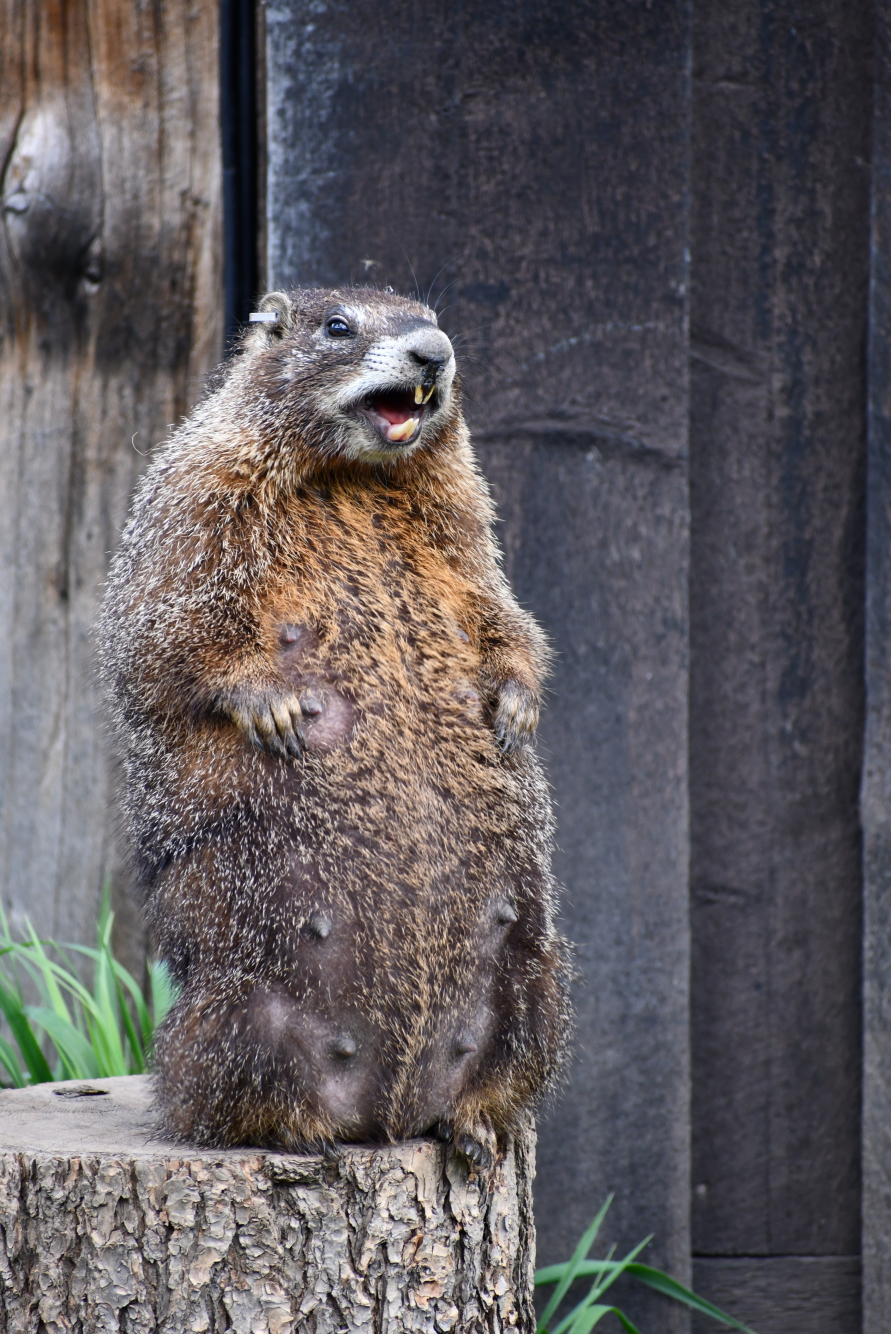
An adult female yellow-bellied marmot with recently emerged young emitting an alarm call in reaction to a red fox (*Vulpes vulpes*) in the Gothic Townsite colony at the Rocky Mountain Biological Laboratory. Red foxes are a major predator of marmot pups. Photo credit: Daniel T. Blumstein.

The yellow-bellied marmots of the Rocky Mountain Biological Laboratory (RMBL) (figure 1) have been studied along a 5 km stretch of the upper East River Valley since 1962, when the late Kenneth B. Armitage started marking and following individuals [31]. Over the years, it has become a valuable long-term study that has generated insights into the demographic and life-history consequences of a variable environment [32,33] and the longevity and senescence in a natural population [34,35]. The study has also provided insights into the development and maintenance of individual behavioural differences [36] and sociality [37,38], and of anti-predator behaviour including alarm communication [39]. Marmots can be described as harem polygynous and socially variable [31]. Of those young that survive their first hibernation (only about 50% do), almost all males and about half of the females will disperse as yearlings before the emergence of the next litter. Those females that do not disperse are recruited into the matriline. While marmots live in a geographic location that we call a colony, their social groups are variable and a lot of our recent work has focused on understanding the adaptive value of this social variation using social network statistics [40]. Interestingly, an emerging theme from this work seems to be that marmots do not gain many benefits from maintaining strong social relationships; females have reduced reproductive success [41], they are more likely to die over winter [42] and live shorter lives [34]. But, in some circumstances, more social females have enhanced summer survival [43].

Marmots are trapped and observed regularly throughout their lives [31,44]. We aim to catch them within a week of emergence from their natal burrow. When captured, we apply numbered ear tags and mark their dorsal pelage with a permanent fur dye (Nyanzol black). We trap each colony location every other week. We collect a variety of samples (hair, blood, faeces) and record any alarm calls emitted when trapped.

Marmots fall prey to a variety of terrestrial (bears—*Ursus americana*, red foxes—*Vulpes vulpes*, coyotes—*Canis latrans*, badgers—*Taxidia taxus*) and aerial predators (golden eagles—*Aquila chrysaetos* and other hawks—*Buteo* spp.) [45]. Red foxes are particularly good at killing pups, but they less frequently kill adults. Domestic dogs have been observed killing marmots too.

**Figure 2.**
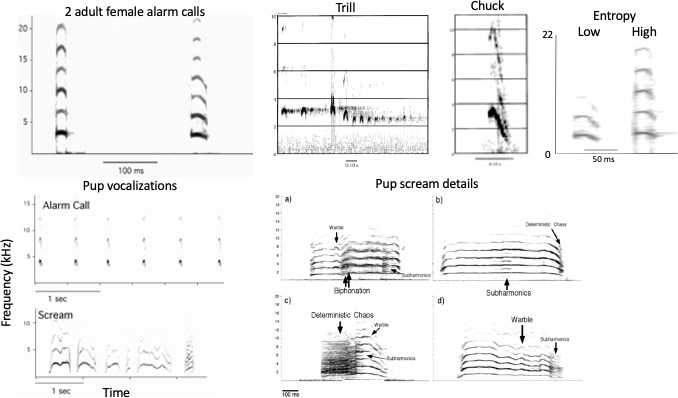
Composite figure illustrating yellow-bellied marmot predator-elicited vocalizations. In all cases, the *x*-axes plot time and the *y*-axes plot frequency; note the different scales to illustrate key features. *Top row from left*: two adult female alarm calls (note individual differences), a trill, a chuck and a low-entropy and high-entropy call that have similar goodness of pitch. *Bottom row from left*: the alarm call from a pup and an example of a pup scream; different types of NLP identified in pup screams. Note that warbles illustrate abrupt frequency jumps. Modified, in part, from images originally in [14,36,46].

In response to encountering a predator, marmots may emit four types of vocalizations (figure 2; [15,47,48]). The most common alarm calls are referred to as chirps or whistles, which for the rest of this article will be simply referred to as alarm calls. The calls are broad-spectrum vocalizations that last for *ca* 50 ms and are often repeated. Bouts of these calls can go on for tens of minutes, and marmots can emit hundreds (and sometimes, thousands) of calls. The calling rate is associated with risk; marmots emit calls faster as risk increases, and the pace of calling slows as risk decreases [48]. Very high-risk situations (an attacking coyote or an eagle) may elicit one or a few chirps before the caller dives into a burrow. Calls are structured and contain stacked harmonics. They also may contain what appears to be deterministic chaos—which looks, on a spectrogram, like white noise.

In very high-risk situations, marmots may trill as they go out of sight into their burrow. These trills are rare, are composed of a series of rapidly paced chirps and are almost always elicited by terrestrial predators (badgers, foxes or coyotes).

In very rare instances, we hear marmots emit chucks [48]. Chucks are low-frequency, very quiet vocalizations and are emitted after a threat has disappeared. They are difficult to record because they are so quiet, but when we see a marmot opening its mouth and shaking its body long after a predatory threat has abated and following a bout of alarm calls, we infer that the marmot is chucking. It is questionable whether this quiet and rare vocalization is an alarm call, but I include it here to be complete. Chucks seem to be less structured and somewhat ‘noisy’, but that could also reflect the relatively low signal-to-noise ratio in the chuck recordings that I have been able to capture.

Finally, young marmots may scream [15]. On average, screams are only produced within the first 7 days after they emerge from their natal burrow. Young marmots can produce alarm calls on the day they emerge and they may scream on the day that they emerge. But after a week or so, they no longer scream, although they may continue to call throughout their lives. Screams, unlike alarm calls, are also sometimes produced when we hold young marmots. These screams are substantially longer than alarm calls and they contain a variety of nonlinear acoustic elements that typically include what appears to be deterministic chaos, rapid frequency shifts up and down, warbles, biphonation and subharmonics.

While my original work focused on vocalizations recorded exclusively at a distance from vocalizing marmots, these calls are of relatively lower quality in that they have relatively low signal-to-noise ratios. Trap-elicited vocalizations are of much higher quality. For all intents and purposes, trap-elicited calls sound identical to calls recorded in the wild. Individuals vary in their propensity to emit alarm calls, but those that are more likely to call in response to a real predator are also more likely to call in response to an approaching person and they are also more likely to emit a call when trapped. We have learnt much from this ‘trap calling assay’.

All age and sex classes may call, but females with young are much more likely to call when their pups are above ground [49]. This is most consistent with calls having a maternal care function, and we have suggested that unlike Belding’s ground squirrels (*Urocitellus beldingi*) and other species that seem to emit calls in ways that are consistent with individuals trying to gain inclusive fitness by warning indirect relatives [4], yellow-bellied marmots call mostly to warn their descendent kin.

Calls are individualistic [46], and marmots care about this individuality [50]. We successfully trained individual marmots to differentiate a reliable caller (i.e. one whose calls were associated with the presence of a badger taxidermic mount) from an unreliable caller (i.e. one whose calls were not associated with the presence of a predator). Compared with a baseline period, marmots hearing reliable callers foraged more after hearing the reliable caller. This was somewhat unexpected given that studies of some primates [51–54] as well as other ground-dwelling sciurid rodents [55–57] found that individuals foraged less or looked more after hearing calls from reliable individuals. Nevertheless, it illustrates that marmots can learn about caller identity, and this and additional findings from Blumstein *et al.* [50] are consistent with the hypothesis that receivers are paying attention to caller reliability. Specifically, we found that marmots were particularly sensitive to unreliable information—they spent more time suppressing their foraging after hearing degraded calls that simulated a distant caller. Hearing a call from a distant caller should create some uncertainty—is the caller signalling about something near them or is it further away from them? If it is further away, could it be near the individual hearing the call and thus be a relatively risky situation, or is the stimulus eliciting the call even further away and thus does not create a risky situation for the perceiver?

Individuals are more likely to call in situations where they are safe. Using a recording array, we were able to locate the caller with respect to its burrow and found that marmots almost always called when very close to their burrows [58]. Indeed, we observed marmots returning to their burrow before calling. This contrasts with Belding’s ground squirrels, which may emit calls in response to aerial predators while running back to their burrows [3]. From this, we infer that yellow-bellied marmots are trying to reduce the direct risks associated with calling. Yet, they are not fully able to do so. Recent work shows that callers that have a greater propensity to emit alarm calls are more likely to die during the summer and live shorter lives [59]. Calling, while potentially life-saving in the short run, is costly in the long run!

## 3. Insights into the production and perception of nonlinear phenomena

We have quantified nonlinearities first in screams and then later in alarm calls (figure 2). We described the suite of NLP in screams and conducted playback experiments that showed that screams frequently had deterministic chaos: 55% contained subharmonics, 13% contained biophonation and 74% had warbles, and these screams were highly individualistic [15]. Because non-scream alarm calls also contain what appears to be ‘noisy’ deterministic chaos, we have quantified this by calculating call entropy [60]. What potentiates noisy alarm calls? A set of analyses has shown that marmots produce noisy calls in at least two situations that are consistent with variations in perceived risk.

The first study looked at the caller’s social network position [61]. We assumed that isolated marmots would be more vulnerable since living socially can be generally viewed as an effective anti-predator strategy. We calculated a number of social network statistics—all of which quantified the centrality or connectedness of an individual in their group. We calculated social network position using the total set of observed affiliative interactions collected during the year for which we had alarm call recordings. We focused on affiliative relationships because these were the most common social interactions [62] and because affiliative and agonistic networks have different functions in our system [63]. We hypothesized that if more connected or central marmots were more secure, they would produce calls with less entropy—our measure of NLP [61]. We also quantified goodness of pitch—a measure of how structured the calls were. These analyses focused on trap-elicited calls because they were of sufficient quality to make detailed measurements. We found that more socially isolated individuals (which we quantified by calculating out-strength—a network measure that quantifies relationships by calculating the number of times individuals initiate interactions with others) produced calls with higher entropy and higher goodness of pitch, which is consistent in that they are producing both noisier and potentially louder calls [61]. Alarm calls might be directed either to predators or conspecifics or to both. Ensuring that the predators hear the calls so that they give up the hunt (e.g. [5]) could account for the more detectable structure. It is also possible that isolated marmots are trying to increase their social status with others by alarm calling because calls are individually distinctive and perceivers track caller identity. Additionally, more connected marmots with higher outcloseness (a network measure that quantifies the number of steps to reach others in the network) produced more structured calls, which we inferred might be associated with them warning individuals to which they were well connected Regardless of the precise function, this study showed us that call structure, and specifically the presence of NLP, was likely modulated by network position in ways that are consistent with the nonlinearity and fear hypothesis, which expects there to be a relationship between acoustic structure and arousal.

The second study looked at health status. Marmots infected with parasites also produced calls with more NLP [64]. We studied the condition dependence of alarm call structure in two ways. First, we asked whether calls from marmots infected with *Eimeria*, an intestinal parasite that consumes energy and presumably increases vulnerability to predators, had noisier calls. Second, we asked if calls from marmots with relatively high neutrophil (N): lymphocyte (L) ratios had noisier calls because these individuals were likely infected and were therefore potentially more vulnerable. We found that there was no significant association between the N : L ratio, but that marmots that were infected with *Eimeria* emitted noisier calls. Since we do not believe that infection with an intestinal parasite directly influences the larynx, we suggested that infection, which could increase an individual’s vulnerability to predators, was associated with producing relatively noisy calls. This result also suggested that marmots can estimate the caller’s relative health status by listening carefully to their calls.

While the above analyses are broadly supportive of the nonlinearity and fear hypothesis, the results of an analysis looking at callers' glucocorticoid levels add complexity. When we trapped a marmot, we collected a faecal sample if the marmot defecated. Using a validated assay [65], we quantified glucocorticoid metabolites. The levels we measured are associated with physiological stress levels experienced the day before; nevertheless, we view these as baseline levels. We also know that marmots that emit calls are more likely to have relatively higher faecal glucocorticoid metabolite (FGM) levels [66]. By looking at paired samples where we had a faecal sample for marmots that were observed calling, we found that marmots with higher levels of FGM had more structured and less noisy calls. Specifically, these calls had higher goodness of pitch and less noise. From this, we concluded that scared marmots produce less, not more, noisy calls [60]. This is consistent with perceived risk-influencing call structure, but not in ways that are entirely consistent with the nonlinearity and fear hypothesis.

Despite this perplexing result, noise matters to perceivers. We conducted a variety of playback experiments to understand the function of alarm calls and specifically nonlinearities. The basic playback experiment was conducted by placing a handful of a favoured food (Omalene 300 Purina horse feed) within 1 m of an active burrow. A speaker was hidden within 10 m of that burrow, and when a marmot emerged and began to forage, we broadcast an alarm call or other control sound. A baseline period before the playback and the response to the playback were videotaped. The change in time allocated to foraging was the most sensitive assay, but we also quantified the time allocated to vigilance, which is traded off against foraging.

Rather than conducting a set of playback experiments that capitalized on natural variation in the amount of noise contained in the calls (as was done with meerkats [67]), we (rather crudely, contra newer technology that is now available [65,66]) added either 10 ms of noise or 10 ms of silence in the same central location of normal alarm calls and compared the listeners' responses between the edited and unmanipulated calls [16]. There was no difference in the responses to either an unmanipulated call or a call with silence added, but adding a burst of noise to the call added reduced the time that marmots allocated to foraging following playback. This strongly suggests that the presence of noise modifies the perception of risk in perceivers in ways that increase their arousal.

More recent work quantified the heritability of NLP in marmot alarm calls. This is an important question because individuals vary in their perceptions of risk, and it is possible that call structure is associated with this. If so, perceivers could assess the relative reliability of callers by listening for noise in their calls—a mechanism not recognized in previous studies that we conducted [68,69]. Again, quantifying noisiness by calculating entropy, we estimated the heritability of noisiness in marmot alarm calls to be about 8.6% (0.001–0.283 95% HDP; [70]). This modest heritable variation suggests that call noisiness is not simply a function of current context, but that it has been subject to selection and, importantly, can further evolve through natural selection.

## 4. But this is not just a story about marmots…

NLP seemingly evoke specific fearful responses in a variety of species, including those that do not vocalize, suggesting that there is something particularly arousing or fear-inducing about sounds that contain NLP. Colleagues and I have conducted a number of playbacks where we created synthetic nonlinear sounds by comparing the response to a pure tone, one followed by a rapid shift up to another pure tone, a rapid shift down to another pure tone and one followed by a brief bout of white noise [71]. Playbacks of these synthetic nonlinear sounds of great-tailed grackles (*Quiscalus mexicanus*) showed that individuals significantly reduced their ‘relaxed behaviour’ after hearing the synthetic NLP compared with control sounds. Similarly, mountain white-crowned sparrows (*Zonotrichia leucophrys*) significantly reduced their relaxed behaviour after hearing the synthetic calls with noise or those that had the rapid frequency shift down [72]. And a non-vocal skink (*Emoia cyanura*) increased rates of looking after hearing a jump-down stimulus compared with controls [73]. But could NLP be associated with fear in humans? To address this, colleagues and I conducted two studies to determine whether NLP are associated with fear in humans.

The first study was correlative and asked whether there were specific associations between NLP and film soundtracks from different genera [74]. If NLP were associated with fear, we expected that iconographic scenes in horror films would have more NLP (particularly noisy NLP) than expected, and we would not necessarily see this association in other genera. Quantifying this took a while. First, we had to convince ourselves that we could objectively quantify NLP in film soundtracks. Since a soundtrack contains a variety of acoustic inputs—human vocal sounds, diegetic sounds (background), instruments and sound effects—a soundtrack is not a natural system that can be overblown. Rather, a soundtrack is a simulated system. By examining spectrograms closely, we trained ourselves to consistently quantify the presence of rapid frequency changes (shift up or shift down), simulated biphonation, simulated subharmonics and simulated noise. We found that iconographic scenes in scary movies (e.g. the shower scene in the film *Psycho*) were more likely to have noisy screams associated with them than what would be expected by chance. We also found that iconographic sad scenes in dramatic films (e.g. the walk to the execution chamber in the film *The Green Mile*) were less likely to have noise in them than expected by chance. This suggests that soundtracks manipulate noise to influence emotions—a hypothesis we directly tested with an experiment.

By creating simple, 10 s films with a change at the 5 s mark (a person walking and making a turn, a person reading and turning a page, a person listening to their phone ring and answering it, etc.) and combining this with music that either changed in predictable ways or not at the 5 s mark, we created a multimodal experiment [75]. The music was meant to be simple melodic sounds that, at 5 s added noise or rapid frequency jumps up or down. According to the motivation-structural rule hypothesis [76], jump-ups might be associated with a fearful or submissive sound, while a threat was associated with a rapid downshift in frequency. Noise would be predicted to be perceived as scary. We asked people to judge these sounds, the videos or the sounds with the videos along two emotional axes: their valence and their arousal.

Surprisingly, we found no significant associations when we analysed the full multi-modal experiment. Perhaps this was because, by design, our videos were rather benign. However, when we looked at humans’ responses to the sounds alone, we found that the addition of ‘noisy’ NLP to an otherwise ‘benign’ bout of music was associated with an increased perception of arousal and decreased valence in human listeners [75]. Unpublished work (G.A. Bryand and D.T. Blumstein) suggests that humans have a specific autonomic response to these sounds.

## 5. Conclusions

Are NLP the sound of fear? By studying first marmots, then birds, lizards and humans, my colleagues and I have shown that noisy sounds, in particular, as well as sounds with rapid frequency shifts down, are particularly evocative for a variety of species. A growing body of empirical work in a variety of species (reviewed in §1) further supports the hypothesis that NLP communicate emotional state. These emotionally evocative signals may be produced intentionally to elicit responses in perceivers, or they may be produced more ‘honestly’, stemming from a loss of vocal control during sound production [20]. In humans, NLP are perceived as arousing and have a negative valence, and a growing body of literature has shown that humans are able to make these assessments from both human and non-human sounds. Arnal *et al*. [77], using playbacks to humans during fMRI monitoring, showed that roughness in sounds (which should be correlated with noise in screams) specifically elicits activity in the amygdala—a key part of the brain involved in processing fear. And, writing in this issue, Arnal & Gonçalves [78] argue that the response to these nonlinear sounds is both functional and has a long evolutionary history. While we have not (yet) conducted similar experiments with marmots using noise or downward-shifting NLP, it seems likely that such experiments would also find associations between these sounds and increased amygdala activity. For all of these reasons, I hypothesize that NLP play an important role in communicating fear. The sound of fear is seemingly nonlinear!

## Data Availability

This article has no additional data.
